# Lack of association between peroxisome proliferator-activated receptors alpha and gamma2 polymorphisms and progressive liver damage in patients with non-alcoholic fatty liver disease: a case control study

**DOI:** 10.1186/1471-230X-10-102

**Published:** 2010-09-08

**Authors:** Paola Dongiovanni, Raffaela Rametta, Anna Ludovica Fracanzani, Luca Benedan, Vittorio Borroni, Paolo Maggioni, Marco Maggioni, Silvia Fargion, Luca Valenti

**Affiliations:** 1 Metabolic Liver Diseases Research Center, Department of Internal Medicine, Università degli Studi di Milano, Fondazione Ospedale Policlinico "Ca' Granda" IRCCS, Milano, Italy; 2 Department of Pathology, Fondazione Ospedale Policlinico Ca' Granda ed Ospedale San Paolo IRCCS, Milano, Italy

## Abstract

**Background:**

Peroxisome proliferator-activated receptors (PPARs) play key roles in the pathogenesis of nonalcoholic fatty liver disease (NAFLD).

**Aim:**

to assess the effect of functional single nucleotide polymorphisms (SNPs) of *PPARα *and *PPARγ2*, previously associated with insulin resistance and dyslipidemia, on liver damage in NAFLD, whose progression is influenced by metabolic abnormalities and inherited factors.

**Methods:**

The Leu162Val *PPARα *and Pro12Ala *PPARγ2 *SNPs were evaluated by restriction analysis. We considered 202 Italian patients with biopsy-proven NAFLD.

**Results:**

The frequency of the evaluated SNPs did not differ between patients and 346 healthy controls. The presence of the *PPARα *162Val allele (prevalence 57%), but not of the *PPARγ2 *12Ala allele (prevalence 18%), was associated with higher insulin resistance (HOMA-IR index 4.71 ± 3.8 vs. 3.58 ± 2.7, p = 0.026), but not with hyperglycemia. The *PPARα *162Val and *PPARγ2 *12Ala alleles were not associated with the severity of steatosis, necroinflammation, or fibrosis.

**Conclusions:**

The presence of the *PPARα *162Val allele was associated with insulin resistance, but not with liver damage in NAFLD. Because of the limited power of the present sample, larger studies are needed to exclude a minor effect of the *PPARγ2 *12Ala allele on necroinflammation/fibrosis in NAFLD.

## Background

Nonalcoholic fatty liver disease (NAFLD), affecting 20-34% of the US and European populations [1,2], is considered the hepatic manifestation of the metabolic syndrome. NAFLD is characterized by hepatic insulin resistance [3], dyslipidemia [4], and is associated with increased mortality due to cardiovascular and liver diseases [5,6].

Liver damage progression occurs when fatty liver is complicated by steatohepatitis (NASH) [5,7,8], which is thought to be provoked by peripheral insulin resistance leading to an increased supply of free fatty acids (FFAs) to the liver, and resulting in oxidative, endoplasmic reticulum and cytokine mediated stress [9].

Inherited factors play a major role in the susceptibility to NAFLD. Indeed, 1) predisposition to NASH appears to cluster with that for metabolic risk factors within families, 2) ethnic differences, partially explained by a different prevalence of patatin-like phospholipase domain-contain 3 protein (*PNPLA3*) variants, have been reported [10-12], and 3) studies in twins demonstrated a high heritability of liver enzymes values reflecting liver fat content [13]. In addition, single nucleotide polymorphisms (SNPs) in genes involved in inflammation, oxidative stress, and fibrogenesis have been associated with the severity of NAFLD [14-19], and recently it has been demonstrated that SNPs influencing Insulin receptor activity predispose to liver damage in NAFLD, confirming that insulin resistance has a causative role in the progression of liver disease [20].

Peroxisome proliferator-activated receptor-alpha (PPARα) is a member of the nuclear hormone receptor superfamily, molecular target of long chain fatty acids, eicosanoids and fibrates (22), and it is expressed at high levels in tissues that catabolize fatty acids such as the liver, skeletal muscles, and the heart. PPARα agonists lower plasma lipid levels, decrease intrahepatic and muscle lipid accumulation, normalize glucose and insulin levels, and reduce the risk of type 2 diabetes in rodent models [21-23]. It has been reported that PPARα downregulation is involved in NASH pathogenesis by reducing FFAs catabolism [24]. Furthermore, the PPARα agonists gemfibrozil and fenofibrate improve dyslipidemia and insulin sensitivity in humans [25]. Preliminary results indicate that another SNP in the PPARα gene, the Val227Ala substitution, may also been implicated in the pathogenesis of NAFLD and could play a protective role against the development of obesity [26]. Since increased hepatic fat, the pathological hallmark of NAFLD, negatively influences insulin signaling [27] and is associated with activation of fibrogenesis [28], we hypothesized that the Leu162Val variant of *PPARα*, previously associated with decreased hepatic lipolysis, dyslipidemia and type 2 diabetes (table 1), could influence liver damage in NAFLD [29].

**Table 1:** Metabolic pathways regulated by the considered genes, effect of polymorphisms on protein function and metabolic parameters.

Gene	Function	Polymorphism	Effect	Previous association
PPARα	Induces lipolysis	Lys162Val (rs1800206)	-	Insulin resistance Dyslipidemia Diabetes [29]
PPARγ2	Induces lipogenesis in adipose tissue	Pro12Ala (rs1801282)	-	Insulin sensitivity [32,40]

-: loss-of-function allele

Peroxisome proliferator-activated receptor-gamma (PPARγ), the molecular target of glitazones, is mainly known to regulate adipocytes differentiation and FFAs uptake and storage, and the PPARγ2 isoform is highly expressed in adipose tissue. PPARγ activation promotes adipogenesis, fatty-acid storage, and eventually obesity, insulin resistance, and type 2 diabetes. Pharmacological activation of PPARγ ameliorates insulin resistance in diabetes, and has been reported to decrease liver damage in NAFLD by restoring adipose tissue insulin sensitivity, thus decreasing FFAs flux to the liver [30,31]. The Pro12Ala SNP of *PPARγ2*, which has been suggest to induce a modest impairment of transcriptional activation due to decreased DNA-binding affinity (table 1), was associated with decreased PPARγ activity in adipose tissue, and decreased insulin resistance and diabetes in Caucasians [32]. However, the effect of the Pro12Ala polymorphism on the risk of diabetes is still controversial [33]. As pharmacological modulation of PPARγ showed promising results on steatosis and necroinflammation in most, but not all, patients with NAFLD, whereas the effect on fibrosis is still undefined [30,34], we hypothesized that functional genetic variants may influence disease progression. In addition, the evaluation of the effect of inherited variability of *PPARγ2 *on liver damage progression may shed light on the possible long-term effects of treatment with PPARγ agonists on liver fibrosis in NAFLD.

Aim of this study was to assess the effect of these well-characterized common functional *PPARα *Leu162Val and *PPARγ2 *Pro12Ala SNPs on the severity of liver disease in a series of Italian patients with biopsy proven NAFLD.

## Methods

### Subjects

Informed written consent was obtained from each patient and control subject, and the study conforms to the ethical guidelines of the declaration of Helsinki.

Two hundred two consecutive Italian unrelated patients from Northern Italy with histologically proven NAFLD and available DNA samples, diagnosed between January 1999 and January 2007 were included. Other causes of liver disease were excluded, including increased alcohol intake (> 30/20 g/day for M/F) [35], as confirmed by at least one family member and carboxydesialylated transferrin determination, HBV and HCV chronic viral hepatitis, autoimmune hepatitis, hereditary hemochromatosis, α1-antitrypsin deficiency, Wilson's disease, and drug induced liver disease. Metabolic parameters and fasting insulin levels, as determined by RIA, were available for each patient. Demographic and clinical features available are shown in table 2.

**Table 2:** Demographic and clinical features of patients with NAFLD and controls.

	Italian controls	Italian adult NAFLD
**number**	**346**	**202**

Sex F	74 (21)	41 (20)
Age years	47.7 ± 12	47.4 ± 11
BMI Kg/m^2^	25.1 ± 2.6*	27.4 ± 3.8*
Total cholesterol mg/dl	194 ± 34*	211 ± 43*
HDL cholesterol mg/dl	56.2 ± 13*	45.1 ± 12*
Triglycerides mg/dl	89 ± 43*	158 ± 91*
Fasting insulin IU/ml	13.4 ± 7*	16.8 ± 11*
Glucose mg/dl	88.5 ± 10*	98 ± 24*
HOMA-IR	2.9 ± 1.6*	4.1 ± 3.3*
ALT UI/ml	23 ± 9*	65 ± 46*
GGT UI/ml	23 ± 16*	87 ± 96*
Fibrosis stage F0/F1/F2/F3/F4	-	104/61/23/8/6 (52/30/11/4/3)

* p < 0.0001 between Italian patients and controls, ():% values

The control group included 346 Italian subjects out of a larger series of 482 blood donors (71%) of the same geographical origin of patients without clinical and biochemical evidence of liver and metabolic disease and no alcohol abuse. We excluded subjects with ALT > 30/18 IU/ml in males/females [36], GGT > 35 IU/ml, BMI > 28, abdominal circumference > 100 cm, glucose levels ≥ 100 mg/dl, triglycerides ≥ 150 mg/dl, or a fatty liver index > 35, which has high specificity to rule out NAFLD in the general population [37]. Fasting insulin levels were available for 259 subjects.

### Histological assessment

Tissue sections were stained with hematoxylin and eosin, impregnated with silver for reticulin framework, and stained with Periodic Acid-Schiff (PAS) for glycogen, Periodic Acid-Schiff diastase (DiPAS) for nonglycogen proteins, Perls for iron, and trichrome for collagen. A single expert pathologist unaware of clinical and genetic data reviewed biopsies. The presence and severity of NASH was assessed according to Kleiner et al. [38]. Briefly, histological activity (NAS: NAFLD activity score) is graded based on steatosis (0-3), lobular inflammation (0-3), and hepatocyte ballooning (0-2), whereas liver fibrosis is staged from 0 (absent) to 4 (cirrhosis). The minimum biopsy size was 1.7 cm and the number of portal areas 10.

We chose as main outcome a fibrosis cut-off of stage > 1 according to Kleiner et al. [38], in the attempt to identify patients with potentially progressive disease at an early stage [39].

### Genetic analysis

DNA was extracted from peripheral blood by the phenol-chloroform method. Success rate in extracting DNA was 100% for each study group. *PPARα *Leu162Val (rs1800206) and *PPARγ2 *Pro12Ala (rs1805192) SNPs were determined by restriction analysis, as previously described [29,40] by personnel unaware of the subjects' clinical status. Moreover, the presence of specific polymorphic alleles as detected by restriction analysis was confirmed in each case by sequencing of random samples of patients. Samples from both NAFLD patients and controls have been included in each batch analyzed, and quality controls were performed to verify the reproducibility of the results. Valid genotypic data were obtained for > 99% of subjects analyzed. Subjects for whom incomplete clinical or genetic data were available were excluded from the analyses.

### Statistical analysis

The sample size was calculated on the basis of the expected relative risk of the presence of a minor allele versus the wild-type allele, the desired power, and significance (p < 0.05, two-tailed).

The sample size had a 99% and 75% power of detecting an OR of 1.5 for NAFLD for the presence of the *PPARα *Leu162Val (rs1800206) and *PPARγ2 *Pro12Ala (rs1805192) SNPs, respectively, and a 100% and 83% power of detecting an OR of 2.0 for fibrosis > 1 in patients with NAFLD, according to the observed genotype frequencies and liver damage distribution, for the presence of the *PPARα *Leu162Val (rs1800206) and *PPARγ2 *Pro12Ala (rs1805192) SNPs, respectively (76% and 33% power of detecting an OR of 1.5). Results are expressed as means ± standard deviation and considered significant when p < 0.05 (two-tailed). Mean values were compared by t-test and by Wilcoxon test, as required. Frequencies were compared by Fisher's exact test.

To allow comparisons with the previous literature and due to the relatively low frequency of the minor alleles evaluated, the effect of genetic factors was tested under the assumption of a dominant effect. Analyses were carried out with JMP 6.0 statistical analysis software (SAS Institute Inc, Cary, NC).

## Results

### Clinical and genetic features of patients with NAFLD and controls

Despite similar age and sex distribution, as expected by enrolment criteria, patients with NAFLD had higher BMI, total cholesterol and triglycerides, lower HDL cholesterol, and higher insulin and HOMA-R index, ALT and GGT levels than controls (table 2).

The frequency distributions of the evaluated SNPs were not significantly different between Italian patients with NAFLD and controls (table 3). The genotype distribution of the polymorphic alleles was in Hardy-Weinberg equilibrium both in patients and controls.

**Table 3:** Frequency distribution of evaluated gene polymorphisms in 202 Italian patients with NAFLD and 346 controls.

Gene	Genotype	Controls	Patients	P
*PPARα* (rs1800206)	Lys162Lys	137 (40%)	87 (43%)	ns
	Lys162Val	159 (46%)	93 (46%)	
	Val162Val	50 (14%)	22 (11%)	

*PPARγ2* (rs1805192)	Pro12Pro	295 (85%)	166 (82%)	ns
	Pro12Ala	50 (14%)	33 (16%)	
	Ala12Ala	1 (1%)	3 (2%)	

P values are shown if < 0.1.

ns: not significant

### Effect of genetic factors on insulin resistance and metabolic features

The effect of inherited factors on insulin resistance, evaluated by HOMA-R index, the presence of diabetes/IFG, and HDL levels is shown in table 4. The *PPARα *162Val allele was nearly associated with higher HDL levels at univariate (46.1 ± 14 vs. 43 ± 10 mg/dl, p = 0.051), but not at multivariate analysis, and patients positive for this allele had a trend for a higher prevalence of diabetes/IFG compared to those without (25% vs. 16%; p = 0.06). No significant effect on HDL levels and diabetes/IFG was observed for the *PPARγ2 *12Ala allele.

**Table 4:** Metabolic parameters of patients with NAFLD subdivided according to the presence or absence of the *PPAR*α 162Val and *PPARγ2 *12Ala alleles.

		Polymorphic allele		
			
	Gene	Present	Absent	p
	HDL	46.1 ± 14	43 ± 10	0.051
				
*PPARα*	Homa-R	4.5 ± 3.7	3.6 ± 2.7	0.026
	Diabetes/IGT	40 (25)	19 (16)	0.060

	HDL	50.7 ± 14	49.4 ± 14	ns
				
*PPARγ2*	Homa-R	4.1 ± 2.1	4.1 ± 3.5	ns
	Diabetes/IGT	8 (18)	51 (22)	ns

P values are shown if < 0.1.

():% values, ns: not significant.

**Figure 1:**
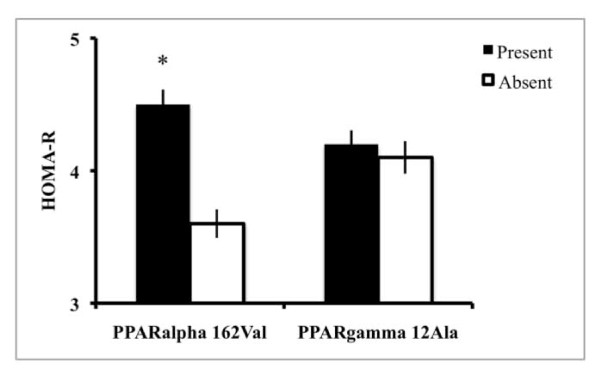
**HOMA-R index in 202 patients with non-alcoholic fatty liver disease (NAFLD) subdivided according to the presence or absence of the *PPARα *162Val and *PPARγ*2 12Ala SNPs**.

In patients with NAFLD, the *PPARα *162Val allele was significantly associated with higher HOMA-R (4.71 ± 3.8 vs. 3.58 ± 2.7, p = 0.026), and the association persisted after the exclusion of 9 diabetic patients treated with metformin (4.8 ± 3.8 vs. 3.6 ± 2.6; p = 0.04). We did not found any association between HOMA-R and the PPARα SNP in controls subjects. In contrast, the *PPARγ2 *12Ala allele had no effect on insulin resistance (Figure 1). The evaluated SNPs were not significantly associated with age, sex, BMI, the presence of hypertension, serum triglycerides, ALT, GGT, and ferritin levels, both in patients and in controls.

### Effect of genetic factors on liver disease severity

**Figure 2:**
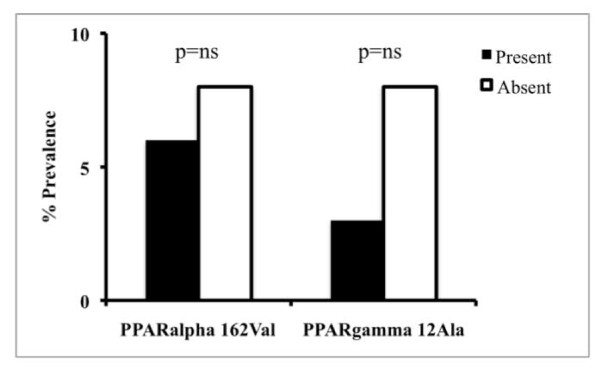
**Prevalence of severe histological activity (NASH activity score (NAS) > 5) in 202 biopsied patients with non-alcoholic fatty liver disease (NAFLD), subdivided according to the presence or absence of the *PPARα *162Val and *PPARγ2 *12Ala SNPs**.

**Figure 3:**
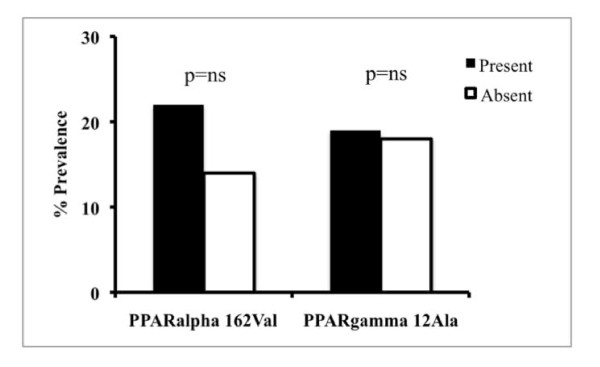
**Prevalence of fibrosis stage > 1 in 202 biopsied patients with non-alcoholic fatty liver disease (NAFLD), subdivided according to the presence or absence of the *PPARα *162Val and *PPARγ2 *12Ala SNPs**.

The effect of inherited factors on the severity of liver disease, evaluated as presence of severe histological activity (NAS > 5) and of severe fibrosis (Kleiner > 1), is shown in figure 2 and 3 respectively. No significant effect of the *PPARα *162Val and *PPARγ2 *12Ala alleles was observed on liver damage. The prevalence of NAS > 5 was similar between patients positive and negative for the *PPARα *162Val allele (6% vs. 8%; p = ns). In patients positive for the *PPARγ2 *12Ala allele, the prevalence of NAS > 5 was lower compared to that observed in negative patients (3% vs. 8%; p = ns). Although the *PPARγ2 *12Ala allele was associated with a lower prevalence of severe histological activity, the sample size is too small to cross the statistical significance threshold. The prevalence of fibrosis > 1 was 22% in patients positive for *PPARα *162Val and 14% in those negative for this allele (p = ns). Also for the *PPARγ2 *SNP, the prevalence of fibrosis > 1 was similar in patients carrying the 12Ala allele compared to the negative ones (19% vs. 18%; p = ns).

Adjustment for age, BMI, ALT levels and diabetes/IGT did not modify the lack of association between the evaluated SNPs and liver damage.

## Discussion

In this paper, we evaluated the effect of well-characterized, functional, common SNPs of genes involved in the regulation of lipid metabolism, and which are amenable of pharmacologic modulation, on the severity of liver injury in patients with NAFLD. Our results, obtained in the largest series of well characterized patients with liver histology available to date, indicate that the *PPARα *162Val allele is associated with insulin resistance but not with liver damage, whereas the *PPARγ2 *12Ala SNP is neither associated with insulin resistance nor with liver damage.

Since NAFLD is considered the hepatic expression of metabolic syndrome, and has a strong genetic component [11,12], we reasoned that genetic factors influencing lipid metabolism might predispose to progressive liver disease in affected subjects [27,29,32]. PPARs, because of 1) their key role in NAFLD pathogenesis 2) genetic variability determining altered function 3) are targets of approved drugs, represent ideal candidate genes.

We preliminarily compared the distribution of PPARs SNPs in Italian patients with NAFLD and healthy controls with normal liver enzymes [36] and absence of metabolic alterations. Even if our sample size had a good power of detecting a 50% increased risk of NAFLD for the presence of the evaluated genetic factors, the prevalence of the SNPs did not differ between patients and controls, suggesting that they do not represent a risk factor for NAFLD. However, we cannot exclude that these genetic variants may influence the risk of NAFLD in patients carrying acquired risk factors, such as obesity, or that other rarer and less characterized variants of these genes may be associated with NAFLD [26].

Indeed, due to the very strict selection criteria our control subjects had no metabolic risk factors and a low fatty liver index, but we did not get direct evidence of the absence of mild fatty liver disease, which may occur even in absence of the metabolic syndrome and abnormal liver function test. Notwithstanding, the main goal of this study was to ascertain whether PPARs SNPs affected the severity of liver disease in patients with biopsy proven NAFLD.

The 162Val *PPARα *SNP is a loss-of-function allele that has been associated with decreased hepatic lipolysis, dyslipidemia and progression of type 2 diabetes (22). Results indicated that the 162Val SNP, present in 57% of the patients, was associated with increased insulin resistance in patients but not in controls, suggesting a possible interaction between liver fat and the *PPARα *162Val allele in the pathogenesis of insulin resistance. Indeed, this genetic variant is supposed to increase insulin resistance and liver fat content by reducing PPARα driven lipolysis, potentially explaining our findings (22). The biological function of this nuclear receptor may also account for the lack of association with hepatic damage despite heightened insulin resistance [41]. Indeed, fatty acid oxidation, contrasted by the 162Val allele, represents a major source of reactive oxygen species that trigger lipoperoxidation in NAFLD [9]. Thus, in patients with the 162Val allele the risk related to increased insulin resistance may be balanced by the protective effect of decreased oxidative stress.

The 12Ala *PPARγ2 *SNP is a loss-of-function allele that been associated with decreased PPARγ activity in adipose tissue, decreased insulin resistance and diabetes in Caucasians [32]. The 12Ala allele, present in 18% of NAFLD patients, was neither associated with liver damage nor with insulin sensitivity. The lack of association between *PPARγ2 *SNP and insulin resistance is possibly related to a prevalent effect of this gene on insulin resistance in tissues different from the liver. We cannot exclude that the evaluated genetic factors may confer a small increase (less than 1.5-fold and 2-fold for the *PPARα *and *PPARγ2 *SNPs, respectively, a threshold for which we had a very good power to detect an association with progressive disease) in the risk of progressive disease, but a larger sample is required to verify this hypothesis. However, the possible effect would be much less strong than that exerted by other genetic variants, such as those influencing insulin receptor signaling and the *PNPLA3 *rs738409 SNP, which were significantly associated with liver fibrosis in the same series [42,43].

We believe that the high relevance of these two PPARs genes for the pathogenesis of liver damage in NASH, and the clinical, although still investigational and off-label, use of PPARγ agonists to treat NASH, requires a deeper knowledge of the relationship between these two genes and NAFLD. Since it is possible that these SNPs may confer a small increase in the risk of NASH progression, which could not be detected by the sample size of this study (this is especially true for the *PPARγ2 *SNP, due to the lower prevalence), we suggest that the issue could be addressed by meta-analysis of the present and future reports. In addition, as we are moving towards individualized medicine, these data could provide the basis to design pharmacogenetic studies to address whether the therapeutic efficacy of PPARγ agonists in patients NASH is affected by the *PPARγ2 *12Ala SNP. Noteworthy, the effect of the Pro12Ala SNP on diabetes risk is still controversial. Indeed, some Authors have reported that, in contrast to previous reports, the Ala12 allele protects against the development of diabetes [44,45], so that the observed trend for a decreased NAS activity in patients carrying the 12Ala allele may reflect a reduced risk for progressive liver damage in subjects carrying this genetic variant, although the difference was not significant due to the insufficient power of the study. This interpretation would be consistent with a protective effect of PPARγ agonists on histological activity, despite the lack of association between the *PPARγ2 *12Ala SNP and insulin resistance in the present series of patients with NAFLD.

## Conclusions

In conclusion, this study indicates that the presence of the *PPARα *162Val and *PPARγ2 *12Ala SNPs influencing lipid metabolism and insulin sensitivity is not a major determinant of progressive liver damage in Caucasian patients with NAFLD. In addition, our data suggest that insulin resistance does not always translate in fibrosis, in that patients carrying the *PPARα *Pro12Ala SNP had increased insulin resistance without different severity of liver damage, and that the genetic background underlying metabolic abnormalities has a major role in determining the clinical outcome [42].

## List of abbreviations

NAFLD: nonalcoholic fatty liver disease; NAS: NAFLD activity score; NASH: nonalcoholic steatohepatitis; SNPs: single nucleotide polymorphisms; PPARs: Peroxisome proliferator-activated receptors.

## Competing interests

The authors declare that they have no competing interests.

## Authors' contributions

PD and LV designed the study, analyzed and interpreted data, and wrote the manuscript, LV and SF funded the study, PD, RR, LB, VB processed the biological samples and performed the genetic analyses, PM and ALF collected and evaluated clinical data, MM reviewed liver biopsies, all Authors read, edited, and approved the final version of the manuscript.

## Authors' information

PD is a senior research fellow and LV assistant professor at the Metabolic Liver Diseases Center, University of Milan, Policlinic Hospital, directed by professor SF. They have contributed with several manuscripts to the field of NASH genetics.

## Pre-publication history

The pre-publication history for this paper can be accessed here:

http://www.biomedcentral.com/1471-230X/10/102/prepub
